# Remote myocardium is also affected in acute myocardial infarction: evidence from advanced CMR relaxometry

**DOI:** 10.1186/1532-429X-18-S1-P219

**Published:** 2016-01-27

**Authors:** Amardeep Ghosh Dastidar, Elisa McAlindon, Jonathan C Rodrigues, Anna Baritussio, Alessandra Scatteia, Estefania De Garate, Chris B Lawton, Chiara Bucciarelli-Ducci

**Affiliations:** grid.410421.20000000403807336NIHR Cardiovascular Biomedical Research Unit, Bristol Heart Institute, Bristol, United Kingdom

## Background

In patients with ST elevation myocardial infarction (STEMI), myocardial tissue injury is not restricted to the territory supplied by the culprit artery but it affects also the remote myocardium supplied by unaffected arteries.(1) The aim was to investigate the T1 and T2 characteristics in infarcted and remote myocardium comparing it with normal reference standard.

## Methods

30 patients (mean age 61 ± 10years and 70% males) with STEMI and successful revascularisation by percutaneous coronary intervention were included. Each subject underwent clinical CMR at 1.5 T with T2 and T1 mapping (MOLLI) pre and post contrast (equilibrium contrast technique for extracellular volume (ECV) quantification) within 48hours of presentation. The T2 and pre and post contrast T1 values were evaluated in each of the 16 AHA myocardial segments.

## Results

Out of 480 myocardial segments, 143 were affected and the rest unaffected or remote. The mean native T1 and the mean ECV in the remote myocardium was higher than the reference standard (1054 ± 65 msec vs 950 ± 21 msec and 0.32 ± 0.06 vs 0.25 ± 0.04). (2) However, the mean native T1 and the mean ECV of the remote myocardium was significantly lower compared to infarcted myocardium (1054 ± 65 msec vs 1153 ± 85 ms, p < 0.0001 and 0.32 ± 0.06 vs 0.46 ± 0.11 ms, p < 0.0001 respectively). In addition the mean T2 of the remote myocardium was significantly lower compared to infarcted myocardium (54 ± 5 vs 63 ± 7 ms, p < 0.0001).

## Conclusions

This is the first study looking at the impact of STEMI on remote myocardium via non-invasive tissue characterisation by advanced CMR relaxometry technique. Our study highlights that the remote myocardium is also affected following STEMI when compared to normal. Albeit the myocardial tissue injury in the infarcted territory is significantly greater than remote myocardium. These findings may have significant future implications in the treatment of STEMI, including targeting the remote myocardium.
